# Mitochondrial genome of *Murina shuipuensis* (Chiroptera: Vespertilionidae) from Shuifu Village, Guizhou, China (type locality)

**DOI:** 10.1080/23802359.2019.1641436

**Published:** 2019-07-16

**Authors:** Zhenglanyi Huang, Yang Yue, Sanjan Thapa, Yifeng Hu, Yi Wu, Wenhua Yu

**Affiliations:** Key Laboratory of Conservation and Application in Biodiversity of South China, School of Life Sciences, Guangzhou University, Guangzhou, PR China

**Keywords:** mitochondrial genome, *Murina shuipuensis*, Chiroptera, Vespertilionidae

## Abstract

*Murina shuipuensis*, a small-sized forest bat with distinct bright orange-yellow ventral fur, is only found from its type locality thus far. In this study, a complete mitochondrial genome of a male individual of *M. shuipuensis* from Shuifu Village, Libo, Guizhou China (type locality) was sequenced using Illumina Hiseq. Total length of its genome is 16,585 bp, including two rRNA genes, 22 tRNA genes, 13 protein-coding genes, and a control region. The general arrangement of genes is similar to the other chiropteran mitochondrial genomes. The phylogenetic inference indicates *M. shuipuensis* and *M. leucogaster* have closer phylogenetic relationship than other taxa that deposited in NCBI-nt. The complete mitochondrial genome provides valuable information for further taxonomic and phylogenetic researches.

*Murina shuipuensis* is a small-sized forest bat with distinct orange-yellow ventral fur (Eger and Lim 2011; Chen et al. 2017) and is discovered from Shuifu village, Guizhou Province, China (type locality). It closely resembles *M. leucogaster* and *M. rongjiangensis* in the shape of skull and in the fur coloration pattern (Eger and Lim 2011; Chen et al. 2017). Although new records of the taxon from nearby provinces are reported (Wang et al. 2016), such specimens may belong to *M. rongjiangensis* rather than *M. shuipuensis* according to our further phylogenetic inference and morphological re-examination (unpublished data). Thus far, such species is only found from its type locality.

In the current study, we determined characteristics of the complete mitochondrial genome (Genbank accession No. MK747249) basing upon an adult male *M. shuipuensis* captured from its type locality (25° 28′ 36″ N, 107° 52′ 45″ E, Elevation 675 m asl). Specimen is stored in the Key Laboratory of Conservation and Application in Biodiversity of South China, School of Life Sciences, Guangzhou University, Guangdong, China (Accession ID GZHU 15619). Total genomic DNA was isolated from muscle tissue using TaKaRa MiniBEST Universal Genomic DNA Extraction Kit, Ltd, Dalian, China. The complete mitochondrial genome was sequencing using Illumina Hiseq sequencing technology. Illumina PE library (460 bp library) was constructed, and the whole genome of mitochondrial was scanned by bioinformatics analysis after quality control of the obtained sequencing data.

Published mitochondrial genome of *M. ussuriensis* (Yoon, et al. 2013), *M. leucogaster* (Yoon and Park 2016), and *M. huttoni* (Zhang, et al. 2016) were used to annotate the mitochondrial genome of *M. shuipuensis*. The mitochondrial genome of *M. shuipuensis* is a circular molecule of 16,585 bp in length, containing 37 genes: two rRNA genes, 22 tRNA genes, 13 proteins genes, and one control region. Most of the genes were encoded on the H-strand while *ND6* (protein-coding gene) and eight tRNA genes (tRNA-Gln, Ala, Aan, Cys, Tyr, Ser, Glu, and Pro) were encoded on the L-strand. Two reading-frame overlaps region were observed between *ATP8* and *ATP6* genes (43 bp shared nucleotides), *ND4L* and *ND4* genes (7 bp shared nucleotides) which were with mutual overlap. The initiation codon of most of the protein-coding genes was ATG, except for those of *ND2* and *ND5* (ATA), *ND3* (ATT). Termination codon of *Cyt b* was AGA, 6 of remaining 12 protein-coding genes were TAA, while the rest were incomplete TA– (*ND1*, *COI* and *COII*) or T–– (*ND2*, *COIII*, *ND4*). Among the 22 tRNA genes with a length ranging from 61 bp (tRNA-ser) to 75 bp (tRNA-leu), all the other 21 tRNA genes can fold into a typical cloverleaf structure, except tRNA-ser. The control region between tRNA-pro and tRNA-phe has a highly repetitive simple sequence of GCATAC.

Altogether 56 released complete mitochondrial genome sequences of Chiropteran species from the GenBank and our sequenced mitochondrial genome were aligned using the MUSCLE (Edgar 2004). Best partitioning scheme with the best likelihood and separated the data sets among genes and nucleotide positions in the different codons were selected using Partitionfinder 1.1.1 (Lanfear et al. 2012). The phylogenetic relationship was inferred by the Maximum-likelihood (ML) method using RAxML V 8.2.4 (Stamatakis 2006, 2014). Our ML tree that reconstructed from complete mitochondrial genome with exclusion of D-loop region, supported all *Murina* taxon clustering together (Figure 1). Compared with other taxa, *M. shuipuensis* and *M. leucogaster* have closer relationship than others (Figure 1). Such phylogenetic result agrees with their morphological similarity in appearance and skull (Eger and Lim 2011). Meanwhile, given its limited distribution and biological information, we hope the mitochondrial genome from this study could benefit further taxonomic and phylogenetic studies, especially on the species and genus *Murina*.

**Figure 1.**
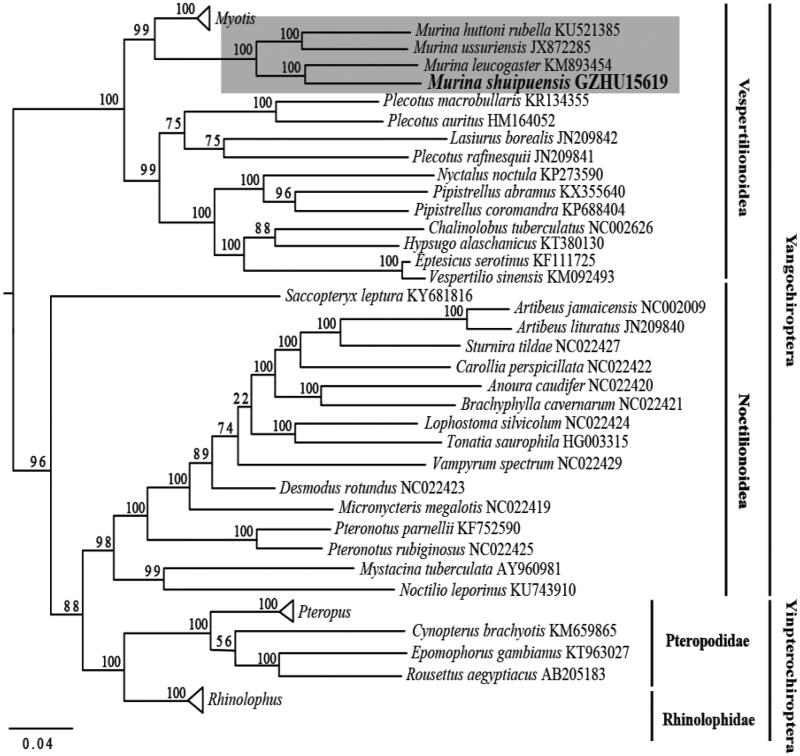
ML phylogenetic trees of 57 chiropteran species based on complete mitochondrial genome. Numbers above the nodes indicate bootstrap values. Branch length is based on ML trees. *Murina* clade is highlighted in shade. Hollow triangles represent clusters of multiple species of *Myotis*, *Pteropus* and *Rhinolophus* genus including 11, 4, and 8 species respectively.
